# Mutations in *TTC19*: expanding the molecular, clinical and biochemical phenotype

**DOI:** 10.1186/s13023-015-0254-5

**Published:** 2015-04-02

**Authors:** Johannes Koch, Peter Freisinger, René G Feichtinger, Franz A Zimmermann, Christian Rauscher, Hans P Wagentristl, Vassiliki Konstantopoulou, Rainer Seidl, Tobias B Haack, Holger Prokisch, Uwe Ahting, Wolfgang Sperl, Johannes A Mayr, Esther M Maier

**Affiliations:** Department of Pediatrics, Paracelsus Medical University Salzburg, Muellner Hauptstr. 48, 5020 Salzburg, Austria; Department of Pediatrics Kreisklinken Reutlingen, Steinenbergstr. 31, 72764 Reutlingen, Germany; Department of Pediatrics, Krankenhaus der Barmherzigen Brueder, Esterhazystr. 26, 7000 Eisenstadt, Austria; Department of Pediatrics, Medical University of Vienna, Waehringer Guertel 18-20, 1090 Vienna, Austria; Institute of Human Genetics, Helmholtz Zentrum München, Ingolstaedter Landstr. 1, 85764 Neuherberg, Germany; Institute of Human Genetics, Klinikum rechts der Isar, Technische Universität München, Trogerstr. 32/3, 81675 Munich, Germany; Present affiliation: Dr. von Hauner Children’s Hospital, University of Munich, Lindwurmstr. 4, 80337 Munich, Germany

**Keywords:** TTC19, Mitochondrial respiratory chain complex III deficiency, Neonatal lactic acidosis, Regression, Loss of speech, Leigh syndrome, Hypertrophic olivary nucleus degeneration, Neurodegenerative disorder

## Abstract

**Background:**

TTC19 deficiency is a progressive neurodegenerative disease associated with isolated mitochondrial respiratory chain (MRC) complex III deficiency and loss-of-function mutations in the *TT19* gene in the few patients reported so far.

**Methods:**

We performed exome sequencing and selective mutational analysis of *TTC19*, respectively, in patients from three unrelated families presenting with initially unspecific clinical signs of muscular hypotonia and global developmental delay followed by regression, ataxia, loss of speech, and rapid neurological deterioration. One patient showed severe lactic acidosis at the neonatal age and during intercurrent illness.

**Results:**

We identified homozygous mutations in all three index cases, in two families novel missense mutations (c.544 T > C/p.Leu185Pro; c.917 T > C/p.Leu324Pro). The younger sister of the severely affected patient 3 showed only mild delay of motor skills and muscular hypotonia so far but is also homozygous for the same mutation. Notably, one patient revealed normal activities of MRC complex III in two independent muscle biopsies. Neuroimaging of the severely affected patients demonstrated lesions in putamen and caudate nuclei, cerebellar atrophy, and the unusual finding of hypertrophic olivary nuclei degeneration. Reviewing the literature revealed striking similarities regarding neuroimaging and clinical course in pediatric patients with TTC19 deficiency: patterns consistent with Leigh or Leigh-like syndrome were found in almost all, hypertrophic olivary nucleus degeneration in all patients reported so far. The clinical course in pediatric patients is characterized by an initially unspecific developmental delay, followed by regression, progressive signs and symptoms of cerebellar, basal ganglia and brainstem affection, especially loss of speech and ataxia. Subsequently, neurological deterioration leading to a vegetative state occurs.

**Conclusions:**

Our findings add to the phenotypic, genetic, and biochemical spectrum of TTC19 deficiency. However, TTC19 deficient patients do show characteristic clinical and neuroimaging features, which may facilitate diagnosis of this yet rare disorder. Normal MRC complex III activity does not exclude the diagnosis.

## Background

*TTC19* (GeneBank: NM_017775; OMIM: 613814) encodes the tetratricopeptide repeat domain 19 consisting of 380 amino acids. The TTC19 protein (NP_060245) has been characterized as a high molecular weight complex, which is embedded in the inner mitochondrial membrane and has been reported to be involved in the assembly and activation of mitochondrial respiratory chain (MRC) complex III [1].

Recently, mutations in *TTC19* have been described in young adults with spinocerebellar ataxia [2-4], a 42-year-old man with rapidly progressive neurological disease [1], and patients with developmental delay in childhood and slowly progressive neurodegenerative disease [1,5,6]. So far, 12 cases are documented in the literature. In all of them, *TTC19* loss-of-function mutations (4 nonsense mutations, 1 deletion of 4 base pairs, 2 duplications of 2 and 17 base pairs, respectively) have been identified leading to premature protein truncation or nonsense-mediated RNA decay.

The biochemical feature indicative for mutations in *TTC19* is an isolated deficiency of mitochondrial respiratory chain (MRC) complex III [1-4,6]. Severe lactic acidosis in blood has not been reported in these patients.

Neuroimaging appears to be quite specific with magnetic resonance (MR) showing T2-weighted signal hyperintensities of caudate nucleus, putamen, and inferior olives in the medulla oblongata as well as atrophy of pons and cerebellum.

We describe the clinical, biochemical, and molecular phenotypes of four pediatric patients with TTC19 deficiency identified by exome sequencing and selective mutation analysis, respectively. We aim to highlight the features of disease manifestation in childhood in order to facilitate diagnosis. For the first time, missense mutations in *TTC19* are reported in patients, as well as a TTC19 deficient patient with a normal activity of the MRC III complex.

## Methods

### Patients

Patients 1 and 2 were recruited at a tertiary university children’s hospital (Paracelsus Medical University, Salzburg), patients 3 and 4 at a regional tertiary referral hospital (Klinikum Reutlingen). Both hospitals are specialized in mitochondrial diseases and are partners of the international MITONET research program.

All clinical data and samples were obtained with written informed consent of the patients’ parents. The ethical committee of the Technische Universität München approved the exome sequencing studies.

### Neuroimaging

MR imaging was performed on a 3-T magnet system (Siemens Healthcare, Erlangen, Germany) and 1.5-T magnet system (GE Healthcare, Reutlingen).

Images were reviewed by a pediatric neuro-radiologist and a pediatric neurologist.

### Exome sequencing and molecular analysis of the TTC19 gene

Total genomic DNA was extracted by standard methods from peripheral blood lymphocytes using standard protocols.

In patients 1 and 2 exome sequencing and variant filtering was essentially performed as described previously using a SureSelect Human All Exon 50 Mb V5 kit (Agilent) for enrichment, and a HiSeq2500 (Illumina) for sequencing (PMID 24461907). Read alignment to the human genome assembly hg19 (UCSC Genome Browser) was done with Burrows-Wheeler Aligner (BWA, v.0.7.5). Detection of genetic variation was performed using SAMtools (v 0.1.18), PINDEL (v 0.2.4 t), and ExomeDepth (v1.0.0). Variant filtering was based on a presumed autosomal-recessive mode of inheritance and focused on homozygous and predictively compound heterozygous rare nonsynonymous variants (MAF < 0.1% in 4,500 control exomes). Identified *TTC19* variants were confirmed by Sanger sequencing.

In family 3 (patients 3 and 4), only sequencing of the *TTC19* gene was performed due to clinical suspicion of TTC19 deficiency. The 10 exons and exon-intron boundaries of *TTC19* (GeneBank: NM_017775; NP_060245; OMIM: 613814) were amplified using intronic primers and subsequently sequenced on the forward and backward strand. All primer sequences are available on request.

### Expression analysis of TTC19

Total cellular RNA was isolated from muscle debris using (TRI-reagent, MRC Inc.). RNA was reversely transcribed with random hexamer primers (Maxima RT, Thermo Scientific) and used for subsequent qPCR analysis. PCR reactions were set up with iQ SYBR Green SuperMix (Biorad) and performed in an iCycler iQ5 (BioRad Laboratories). Three pairs of oligonucleotides were designed to flank either the predicted missense mutation c.554 T > C (p.Leu185Pro) in exon 6 (Exon4-forward 5′-ATACGGGGTCAGCTTGAAAA-3′ and Exon7-reverse 5′-TGCAGAATTCATAGCCAGCA-3′) or the predicted missense mutation c.971 T > C (p.Leu324Pro) in exon 9 (Exon7/8-forward 5′-GACACCCACAGACCATTGTG-3′ and Exon10-reverse 5′-CAGCTTTGCTTGCTTCAGTG-3′). In addition, a third pair of primers from exon 8 to 9 (Exon8-forward 5′-CGAGGCAGAGATCATCCAG-3′ and Exon9-reverse 5′-CCAGGGTAGTAGCCAGGTCA-3′) was designed. Two housekeeping genes HPRT (HPRT-forward 5′-TTCCTTGGTCAGGCAGTATAATC-3′ HPRT-reverse 5′-GGGCATATCCTACAACAAACTTG-3′) and RPL27 (RPL27-froward 5′-GCTGGAATTGACCGCTACC-3′ and RPL27-reverse 5′-TCTCTGAAGACATCCTTATTGACG-3′) were used as controls. All experiments were performed in duplicates. To calculate ∆Ct (difference of cycle thresholds), the mean values of the housekeeping gene reactions were subtracted from the respective TTC19 reactions. The ∆∆Ct value was calculated by subtracting the ∆Ct values of the controls from those of the patients.

### Biochemical studies

Skeletal muscle tissues were homogenized in extraction buffer (20 mM Tris–HCl, pH 7.6, 250 mM sucrose, 40 mM KCl, 2 mM EGTA) and subsequently centrifuged at 600 *g* generating the postnuclear supernatant (600 *g* homogenate), which was used for measurement of MRC enzyme activities and western blot analysis.

MRC enzyme activities were determined as published elsewhere [7-9]. Briefly, rotenone-sensitive complex I activity was measured spectrophotometrically as NADH/decylubiquinone oxireductase. The enzyme activities of citrate synthase, complex IV (ferro-cytochrome c/oxygen oxidoreductase), and the oligomycin-sensitive ATPase activity of the F_1_F_0_ ATP synthase (complex V) were measured in buffer conditions described by Rustin et al. [10]. Succinate dehydrogenase activity (SDH; complex II) was determined according to Rustin et al. with slight modifications [10]. Buffer conditions and procedure of determination of complex III activity can be retrieved in Feichtinger et al [9]. All spectrophotometric measurements were performed at 37°C.

### Immunoblotting

For western blot analysis, a total of 10 μg protein of 600 *g* homogenate was separated on 10% acrylamide/bisacrylamide gels and transferred to nitrocellulose membranes using CAPS buffer. Washing and blocking procedures were performed as previously described [10].

The following primary antibody dilutions, incubation times and temperatures were used: monoclonal rabbit anti-TTC19 (1:1,000; 1 h, room temperature; Abcam), monoclonal mouse anti-UQCRC1 (Core 1) (1:1,000; 1 h, room temperature, MitoSciences), monoclonal mouse anti-UQCRFS1 (1:1,000; 1 h, room temperature; MitoSciences), monoclonal mouse anti-porin (1:2,000, 2 h, room temperature; MitoSciences). After washing, the membranes were incubated with secondary antibodies as follows: UQCRC1, UQCRFS1 and porin with labeled polymer-HRP-antimouse (1:1,000; 1 h, room temperature, EnVision kit, Dako), TTC19 with labeled polymer-HRP-anti-rabbit (1:1,000; 1 h, room temperature, EnVision kit, Dako). Detection was carried out with Lumi-Light^PLUS^POD substrate (Roche).

### BN-PAGE (blue native polyacrylamide gel electrophoresis)

Solubilized mitochondrial membranes were prepared from isolated fibroblast mitochondria as described previously [11]. Briefly, fibroblast mitochondria were sedimented by centrifugation at 13,000 g for 15 min. Membranes were solubilized with 1.5% laurylmaltoside for 15 min and centrifuged for 20 min at 13,000 g. Solubilized membranes were loaded on a 5% to 13% polyacrylamide gradient gel and separated electrophoretically. For immunoblot analysis, membrane preparations were separated by BN-PAGE (5-13%) and blotted onto polyvinylidene difluoride membrane (Hybond-P, GE Healthcare) using a CAPS buffer (10 mmol/l 3-cyclohexylamino-1-propane sulfonic acid pH 11, 10% methanol). The membrane was washed in 100% methanol for 2 min and blocked for 30 min at room temperature in 1% blocking solution (Roche) dissolved in TBS-T. The primary antibodies, diluted in 1% blocking solution-TBS-T, were added 1 h at room temperature. The following primary antibody dilutions were used: complex I subunit NDUFS4 monoclonal antibody (1:1,000; MitoSciences) complex V subunit α monoclonal antibody (1:1,000; MitoSciences) and complex II subunit SDHA monoclonal antibody (1:30,000; MitoSciences). After extensive washing, blots were incubated for 1 h at RT with secondary mouse antibody (1:100; DAKO polymer Envision Staining Kit). Detection was carried out with Lumi-LightPLUS POD substrate (Roche).

## Results

### Clinical findings

Clinical, biochemical, and molecular data of the 4 patients with *TTC19* mutations is summarized in the Table 1.

### Patient 1

Patient 1, a boy, was born by caesarian section to healthy, consanguineous Turkish parents after uneventful pregnancy (birth weight 3620 g, length 50 cm, head circumference 35 cm, APGAR 9/10/10). The boy had four older healthy siblings (1 sister, 3 brothers). He presented with severe lactic acidosis in the neonatal age and during intercurrent infections at the age of 4 months (RSV), 5 months (viral infection), 10 months (tonsillitis), and 16 months (complicated febrile seizure). Lactic acid levels were normal in repeated analyses between the infections. The boy’s psychomotor development was globally delayed. He never walked independently. In his second year of life, regression occurred starting with a loss of speech and vocalizing at age 13 months, massive muscular hypotonia and muscle weakness.

T2-weighted MR imaging at the age of 16 months revealed symmetric hyperintensities of the basal ganglia and the periventricular white matter. Skeletal muscle biopsy showed signs of mitochondrial involvement, e.g. numerous ragged red fibers and low COX reaction, and partially reduced activities of MRC complexes II and III.

**Figure 1:**
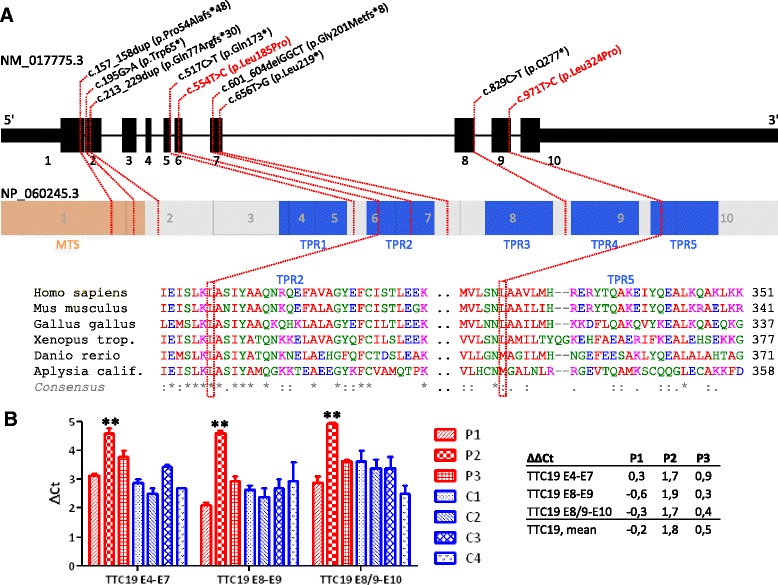
**Mutations in** ***TTC19*** **and expression of TTC19. A**. Novel and reported Mutations in *TTC19* and their phylogenetic conservation. A schematic drawing shows the 10 exons, the mitochondrial targeting sequence (MTS), and the 5 tetratricopeptide repeats (TPR) of *TTC19.* The previously reported mutations are indicated in black, the two novel missense mutations reported here are highlighted in red. Sequence alignment with different species shows the affected amino acid residues to be highly conserved. **B**. TTC19 transcript levels in muscle of patients (P) and controls (C). Normal amounts of TTC19 transcripts compared to controls were found in patients 1 and 3 carrying missense mutations (c.971 T > C; p.Leu324Pro and c.554 T > C; p.Leu185Pro). A significant reduction of TTC19 transcripts was observed in patient 2 who carries the stop mutation c.656 T > G (p.Leu219*). A repeated measures ANOVA and a Tukey post test to compare all pairs of columns was used for statistical analysis (**p < 0.01).

On request of the parents, the boy was referred to a tertiary University hospital for second opinion and further diagnostic work-up at the age of 2 years. A second muscle biopsy was performed revealing normal PDH activity and normal pyruvate oxidation, but significantly decreased isolated MRC complex III deficiency with a residual activity of 28% of the lowest control. The findings of ragged red fibers and low COX reaction were not confirmed. Using exome sequencing, the novel missense mutation c.971 T > C (p.Leu324Pro) was identified in a homozygous state. The mutation affects the conserved tetratricopeptide repeat (TPR) domain 5 (Figure 1A).

**Figure 2:**
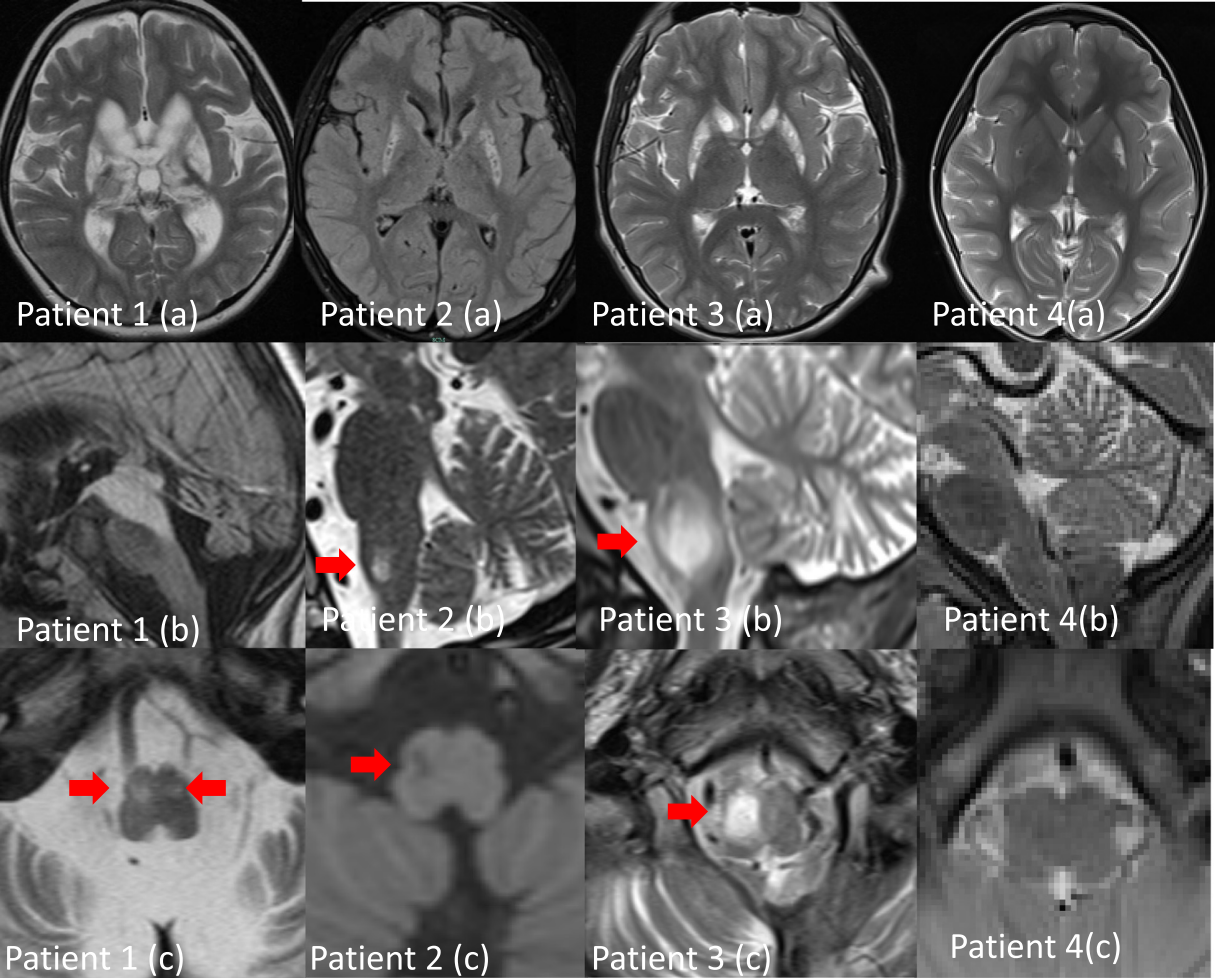
**Brain magnetic resonance imaging of the four patients.** Hyperintensities in T2 (Flair 2a, 1b)-weighted images indicate pathology of basal ganglia (1a, 2a, 3a, 4a), thalamus (1a), and mesencephalon (1b). Cerebellar atrophy is shown on in 1b, 2b, 3b. The increased interfoliar spaces in 4b may be interpreted as early sign of cerebellar atrophy in the clinical context of patient 4. Hypertrophic degeneration of olivary nuclei are marked with red arrows in 1c, 2b and c (T1 signal hypointensity), 3b and c. It is not seen in 4b, c.

MR imaging at the age of 4 years showed a rapid progression with bilateral signal hyperintensities in T2-weighted images of the basal ganglia (putamen, pallidum), thalamus, the mesencephalon, and the medulla oblongata with hypertrophic olivary degeneration as well as severe cerebellar atrophy (Figure 2, column 1).

The boy is now 10 years old. Since the age of 8 years, he is unable to communicate showing only unspecific reactions to pain. He is severely hypotonic and lacks any postural control. He is completely immobile with no spontaneous movements but moderate bilateral spasticity of the limbs. He requires feeding via a gastric tube. His main medical issues are refractory epilepsy with predominantly tonic seizures and recurrent airway infections. Currently, he is treated with antiepileptic drugs and carnitine, supplementation with coenzyme Q_10_ and riboflavin has been ceased.

### Patient 2

Patient 2, a boy, is the only child of a single Austrian mother, who reported tobacco and drug use during pregnancy. The ethnic background of the father is unknown. The boy was born at term (3895 g, 55 cm length, 38 cm head circumference; APGAR 9/10/10) followed by an uneventful postnatal period.

In his 3rd month of life, he presented with an episode suspicious of convulsion, but diagnostic work-up was unremarkable. At the age of 19 months, a global developmental delay regarding motor skills, speech, and social contact was documented. In addition, muscular hypotonia, muscle weakness as well as disturbed coordination were described.

Until the age of 6 years, a constant developmental delay with dyspraxia, dysarthria, and lack of initiative and motivation were noted. Neuroimaging by T2-weighted MR showed bilateral hyperintensities within the putamen, the caudate nucleus and the periaqueductal gray matter of the mesencephalon. At the age of 7 years, he presented with rapidly progressive ataxia, dysarthria, and dysphagia. His deep tendon reflexes were exaggerated, the muscle tone reduced. Laboratory findings including CK, liver parameters, lactic acid, acylcarnitines, and amino acids were normal. MRI of the brain revealed a considerable progression of the pathologies with respect to basal ganglia, mesencephalon, pons, medulla, and cerebellum. T2 weighted images showed signal hyperintensities with “cystic” aspects in caudate nucleus and putamen, increased T2 signaling in the posterior medial thalamus, the mesencephalon with the periaqueductal gray matter, and hypertrophic olivary degeneration of the right more than the left olivary nucleus, as well as cerebellar atrophy (Figure 2, column 2).

Skeletal muscle biopsy and analysis of respiratory chain enzymes indicated isolated deficiency of complex III (31% of lowest control) without signs of mitochondrial disorder in histology and histochemistry. Exome sequencing identified the previously described nonsense mutation c.656 T > G (p.Leu219*) was shown in a homozygous state [1].

The boy is now 9 years old, his clinical condition showing a constant decline. He is wheelchair-bound due to bilateral spasticity and dystonia. Only little activity is remaining in his left arm and hand. He lost speech and is able to communicate only yes and no. Due to severe dysphagia, he requires to be fed via a gastric tube. He is supplemented with antioxidative medication (carnitine, coenzyme Q_10_, thiamine, riboflavin).

### Patient 3

Patient 3, a boy, is the 2nd child of healthy parents. He was born after a normal full-term pregnancy. The postnatal period was unremarkable. The mother grew up in Macedonia, the father in Croatia. Both parents belong to the ethnic group of Romani people. The boy has 3 healthy siblings (16, 2 ½, and 1 years of age). A 7-year-old sister (patient 4) shows mild muscular hypotonia and retardation of motor skills.

Family history was remarkable for deafness of two cousins of the mother, for trisomy 21 of one cousin of the mother, and for a remote male relative showing similar signs and symptoms as patient 3 (no clinical data available).

Since the family lived in several European countries, there was no constant follow-up by a family doctor. According to the parents, the boy’s initial psychomotor development was normal. He started to walk independently at the age of 15 months, however, spoke his first words not before the age of 2 years. At the age of 3 years, he started kindergarten (nursery school), where a delayed statomotor development with frequent stumbling was stated. First grade of primary school had to be repeated two times. At the age of 8 years, neurological deterioration and regression occurred beginning with a loss of speech, followed by disturbed gait and paresis. By the age of 11 years, he had lost almost all of his acquired skills, and was wheelchair-bound. He communicated with his eyes, facial expressions, and little hand movements using a tablet computer.

MR imaging at the age of 9 years revealed hyperintensities of nucleus lenticularis and nucleus caudatus suspicious for mitochondrial disease, a follow-up neuroimaging 2 ½ years later a loss of volume in the putamen, and signs of cerebral atrophy. MR spectroscopy showed a clearly elevated lactic acid peak with reduced NAA. Lactic acid in cerebrospinal fluid (CSF) was normal.

Skeletal muscle biopsy was unremarkable with normal activities of the MRC enzymes and no signs of mitochondrial disease in histology and histochemistry.

Following a non-febrile upper respiratory tract infection at the age of 14 years, the boy developed sepsis and rapidly deteriorated with severe muscular hypotonia, tachycardia, dysphagia, and difficulties in breathing occurred. He was admitted to the intensive care unit of a tertiary hospital specialized on mitochondrial disease for further treatment and diagnostic work-up. No lactic acidosis was reported during this episode.

MR imaging revealed inhomogenous hyperintensities of the striatum (caput nuclei caudate, putamen), hyperintense hypertrophic degeneration of the right inferior olivary nucleus, and cerebellar atrophy (Figure 2, column 3). Analysis of a second muscle biopsy showed unspecific impairment of mitochondrial energy metabolism but no deficiency of MRC complexes.

Due to the MR imaging and lack of lactic acidosis, western blotting of TTC19 protein was performed revealing only traces of TTC19 protein. Subsequent sequencing of *TTC19* showed a novel missense mutation, c.554 T > C (p.Leu185Pro) in a homozygous state. The mutation affects a conserved position in the TPR domain 2 (Figure 1A).

The boy is now 14 years old. He shows severe bilateral spasticity and refractory symptomatic epilepsy. Only little spontaneous movements of the upper extremities are seen. Because of muscular hypotonia and dysphagia, feeding via a PEG tube is required. He is treated with an antiepileptic drug (levetiracetam) and is supplemented with coenzyme Q_10_ and thiamine.

### Patient 4

Patient 4, a girl, is the younger sister of patient 3. She was born after a normal full-term pregnancy (birth weight 3500 g). Her initial psychomotor development was normal, with sitting at the age of 7–8 months, first walking at the age of 13 months, and first words at the age of 9 months. At the age of 6 years, due to muscular hypotrophy of the legs, MR imaging was performed with normal results (no data available).

After her brother’s diagnosis was identified, the girl was re-evalutated. She attends primary school with sufficient success. On neurological examination, she shows muscular hypotonus, brisk tendon reflexes, delayed fine motor skills, but no ataxia.

MR imaging at the age of 7 years showed hyperintensities in caput nucleus caudati and basal parts of the putamen, as well as increased interfoliar spaces in the cerebellum (Figure 2, column 4). This abnormal finding may be interpreted as an early sign of cerebellar atrophy in this clinical context. The inferior olives appear unaffected.

Subsequent sequencing of *TTC19* showed the same novel missense mutation, c.554 T > C (p.Leu185Pro), in a homozygous state as her brother. The girl is supplemented with coenzyme Q_10_, thiamine, and biotin.

#### Biochemical and molecular findings

Exome sequencing and Sanger sequencing, respectively, revealed *TTC19* mutations in all four patients (Table 1). In patients 1 and 2 investigated by exome sequencing, a filter for rare recessive-type nonsynonymous variants identified 28 and 6 genes, respectively. In both cases, *TTC19* was the only gene coding for a protein with a confirmed or predicted mitochondrial localization according to a MitoP2 score >2.5 [12]. All mutations were found in the homozygous state in the patients and confirmed in the heterozygous state in the parents (except for patient 2, whose father is not known to us). Two mutations (c.971 T > C, c.554 T > C) are novel and the first reported pathogenic missense mutations in *TTC19*, whereas the nonsense mutation c.656 T > G has been described previously [1] (Figure 1A). The two missense mutations are not present in >122.000 control alleles of the Exome Aggregation Consortium (ExAC) Browser (Cambridge, MA (URL: http://exac.broadinstitute.org) 12/2014) while the nonsense variant c.656 T > G is listed 5 times in 122.582 alleles, however, only in the heterozygous state. The expression of TTC19 mRNA in muscle was investigated by quantitative real-time PCR and revealed a normal amount of cDNA in case of the two missense mutations (patient 1 and patient 3) (Figure 1B). The size of the products was normal, too (data not shown). In case of patient 2, carrying the nonsense mutation c.656 T > G (p.Leu219*), the amount of cDNA was significantly decreased (ΔΔCt = 1.8) pointing to nonsense-mediated decay of this RNA (Figure 1B).

**Table 1:** **Clinical, biochemical, and molecular findings of the 4 patients with TTC19 deficiency**

		**Patient 1** ^**a**^	**Patient 2** ^**a**^	**Patient 3** ^**b**^	**Patient 4** ^**b**^
**Sex**		male	male	male	female
**Ethnic origin**		Turkish	Austrian	Romani	Romani
**Age at onset of symptoms**		neonatal	19 months	3 years	6 years
**Signs/symptoms at onset**		Lactic acidosis	Developmental delay, hypotonia, dysarthria, ataxia	Developmental delay, hypotonia, regression, ataxia	Mild developmental delay, hypotonia
**Current clinical condition**		10 years, alive, vegetative state, refractory epilepsy, bilateral spasticity	9 years, alive, bilateral spasticity, dystonia, wheelchair-bound, dysarthria, dysphagia	14 years, alive, wheelchair-bound, bilateral spasticity	7 years, motor skills delayed, normal schooling
***TTC19*** **mutations (homozygous)** ^**c**^		c.971T>C; p.Leu324Pro	c.656T>G; p.Leu219*^d^	c.554T>C; p.Leu185Pro	c.554T>C; p.Leu185Pro
**Muscle biopsy**					not performed
MRC complex III activity	% of Lowest Control	28%	31%	normal	
Absolute value (reference range)		0.40 (1.45-3.76)	0.45 (1.45-3.76)	1.49 (1.45-3.76)	
Western blotting	TTC19	trace	trace	trace	
	Complex III, core 1	normal	normal	normal	
	Complex III, core 2	normal	normal	normal	
	Complex III, Rieske	normal	normal	normal	
**Metabolic data**					
Elevated lactate (blood/CSF)		+ / n.a.	+ / n.a.	- / -	-
**Neurological status**					
Ataxia		+	+	+	-
Dysphagia		+	+	+	-
Dysarthria		+	+	+	-
Dystonia		+	+	-	-
Enhanced tendon reflexes		+	+	+	+
Spasticity		+	+	+	-
Hypotonia		+	+	+	+
Symptomatic epilepsy		+	-	+	-
Regression/decline		+	+	+	-
Loss of speech		+	+	+	-
**Neuroimaging features**					
Leigh syndrome		+	+	+	-
Hyperintensities (T2-weighted)	N. caudatus	+	+	+	+
	Putamen	+	+	+	+
	Medulla oblongata	+	+	+	-
	Mesencephalon	+	+	+	-
	Olivary nucleus	+	+	+	-
Leukoencephalopathy		+	+	n.a.	-
Cerebellar atrophy		+	+	+	(+)
Pontine atrophy		-	-	-	-
Cortical atrophy		+	+	+	-
^1^H-MRS (lactate peak)		n.a	n.a	+	n.a.

Abbreviations are as follows: n.a., not available; MR, magnetic resonance; ^1^HMRS, proton magnetic resonance spectroscopy; Enzyme activities were normalized to citrate synthase (CS). Absolute values and reference ranges are given in (mU/mU CS).

^a^Investigated by exome sequencing.

^b^These individuals are siblings.

^c^cDNA (NM_017775);Protein (NP_060245).

^d^Mutation was previously published [1].

**Figure 3:**
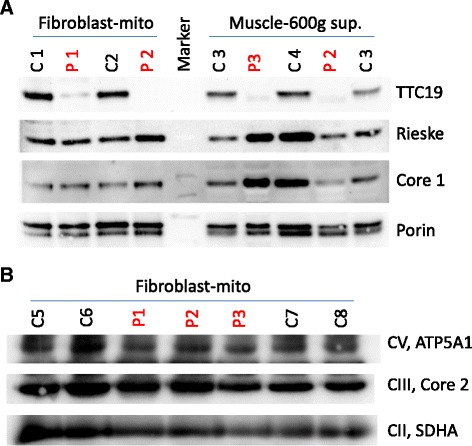
**Western blot analysis of muscle homogenisates and fibroblast mitochondria of TTC19 patients (P) and controls (C). A**. SDS-PAGE: TTC 19 protein in markedly reduced in patient 1 and absent in patients 2 and 3. There is no obvious reduction in core 1 and Rieske protein, both subunits of MRC complex III, in comparison to porin. Porin is a protein of the outer mitochondrial membrane and was used as a loading control. **B**: Blue Native-PAGE: There is no obvious reduction of complex III (CIII, core 2) in patients 1, 2, and 3 compared to controls. Complex II (CII, SDHA) and complex V (CV, ATP5A1) were used as loading controls.

Our patients showed an isolated deficiency of MRC complex III, except for patient 3, who demonstrated normal activities of MRC complex III in two independent muscle biopsies (Table 1). In western blot analysis, TTC19 protein was severely reduced (patient 1, fibroblasts) or not detectable in patients 2 and 3 (skeletal muscle and fibroblasts) (Figure 3A). The additionally tested subunits of complex III, namely core 1 and Rieske protein were detectable in normal amounts, as well as porin (Figure 3A). BN-PAGE experiments in fibroblast mitochondria revealed that complex III is fully assembled in all three patients compared to complex II and complex V (Figure 3B).

## Discussion

Mutations in *TTC19* have been described in 12 patients from eight families so far [1-6]. Seven of these patients showed disease manifestation in adulthood, six of them presenting with progressive cerebellar ataxia and dysarthria, variably accompanied by mental impairment and psychiatric manifestations [2-4]. Another patient showed subacute, rapidly progressing neurodegeneration at the age of 42 years, leading to death within 2 years [1]. Patients with an infancy-onset disease displayed a progressive neurodegenerative disorder [1,6] and global developmental delay followed by language regression [5].

In this report, we describe the clinical, biochemical, and molecular data of four new pediatric patients with TTC19 deficiency from three unrelated families. First clinical features were unspecific in all patients consisting of global developmental delay, delayed motor skills, and muscle hypotonia (Table 1). Subsequently, progressive signs and symptoms of cerebellar, basal ganglia and brainstem affection were observed such as ataxia, dysarthria, aphasia, dysphagia, dystonia and spasticity in the severely affected patients 1, 2, and 3. Neurological deterioration occurred at different progression rates leading to severe motor and cognitive impairment in patients 2 and 3. Patient 1 is in a minimally conscious state, patient 4 presumably in an early stage of the disease.

**Figure 4:**
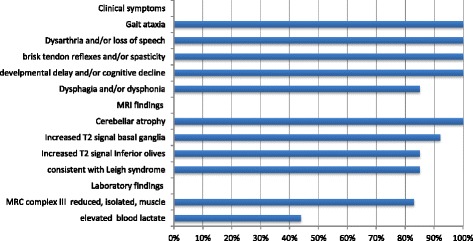
**Main clinical, biochemical, and MRI features in 14 patients with TTC19 deficiency.** The y axis lists the main clinical symptoms, MRI and laboratory findings, the X axis indicates the frequency (%). The figure summarizes 14 patients reported in the literature [1-3,5,6]. Patient 4 of this work is not included, as she is still oligosymptomatic. Remarkably, elevated blood lactate is found in less then fifty percent of patients and isolated MRC complex III deficiency is not a constant finding in all patients.

The clinical course of the patients described herein coincides very well with the disease manifestation of the three patients with an onset of disease in infancy reported by Ghezzi et al. A striking common finding in our patients was regression beginning with an early and pronounced loss of speech in all three severely affected patients 1, 2, and 3. Loss of speech is not a frequent neurological finding in pediatric patients, but may be a characteristic finding for TTC19 deficiency and thus, a hint for diagnosis. It was indicated as major finding in the 4-year old boy described by Atwal et al. as well as in 2 of 3 patients described by Ghezzi et al. In the patient reported by Melchionda et al. it was a rather late symptom. Figure 4 summarizes the major clinical, biochemical, and neuroimaging features of 14 TTC19 patients reported so far [1-3,5,6], which appear to be quite characteristic, to draw the clinicians’ attention to a potential diagnosis of TTC19 deficiency.

Interestingly, patient 1 showed severe lactic acidosis in the neonatal age as well as during intercurrent viral infections thereinafter. Neonatal lactic acidosis is found in several types of mitochondrial disorders [13], but has not been associated with TTC19 deficiency so far. Patients with TTC19 deficiency have been described to show normal or only slightly elevated concentrations of lactic acid in blood [1,2,6].

At the age of 7 years, patient 4 shows only mild and unspecific neurological impairment. She was diagnosed with TTC19 deficiency in the context of her brother’s (patient 3) rapidly deteriorating clinical course. At the moment, it is difficult to predict the onset of further symptoms as well as the rate of progression. Even within the same family, the clinical phenotype has been reported to vary significantly in patients with TTC19 deficiency [3], as in other mitochondrial disorders associated with neurodegeneration [14-16].

MR imaging of our patients revealed symmetrical hyperintense lesions of the basal ganglia, thalami, brainstem, and the cerebellum on T2-weighted images consistent with Leigh syndrome [17] and, thus, led to the diagnostic workup for mitochondrial disease. However, the genetic background of Leigh syndrome is heterogeneous and often remains unsolved despite extensive investigations [16]. In addition to MRI patterns consistent with Leigh syndrome, our severely affected patients 1, 2, and 3 showed hypertrophic olivary degeneration at the progressive state of the disease. Hypertrophic olivary degeneration is a rare finding in pediatric patients [18]. It results from lesions within the dento-rubro-olivary pathway and has been reported in children after tumor surgery in the posterior fossa and the brainstem [19-23]. Furthermore, recent studies suggest, that hypertrophic olivary degeneration in children is associated with mitochondrial disease, especially with Leigh and Leigh-like syndrome [18,24-28]. In a cohort of 125 children with mitochondrial and genetic disorders, hypertrophic olivary degeneration was found in 40% of patients with Leigh or Leigh-like syndrome (10/25), but not in patients with other mitochondrial (0/25) or genetic (0/75) disorders [18]. Mutations in *SURF1* and *POLG* have been identified in patients with mitochondrial disease and hypertrophic olivary degeneration, but are not a consistent finding [18,25,28]. In patients with Leigh syndrome due to mutations others than *SURF1* and *POLG*, hypertrophic olivary degeneration is hardly found [18] with the exception of one case of PDH deficiency [26]. Remarkably, hypertrophic olivary degeneration is a consistent finding in our severely affected patients (Figure 2) and in nine out of ten previously published patients with *TTC19* mutations who underwent MR investigation [1-5]. The patient reported by Melchionda et al. showed no hypertrophic olivary degeneration in neuroimaging that was done at a relatively early stage of the disease. We suggest that hypertrophic olivary degeneration in patients with Leigh or Leigh-like lesions in brain MRI is – in addition to *SURF1* and *POLG* - a strong diagnostic indication for *TTC19* mutations and thus can facilitate diagnostic workup. However, hypertrophic olivary degeneration can be very subtle on MR imaging and may be missed if not intently looked for.

Hypertrophic olivary degeneration has been described to be clinically associated with palatal tremor and myoclonus [28,29], a finding that has not been found in our patients. One might, however, speculate that the clinical signs of dysarthria, loss of speech and dysphagia are consequences of an impaired palatal function in hypertrophic olivary degeneration to some extent. Furthermore, the inferior olivary nucleus has shown to be involved in the pathogenesis of ataxia in mitochondrial disorders and to be vulnerable to energy deprivation with a clinical presentation most often after first year of life [30-32].

Patient 4, the so far mildly affected sister of patient 3, showed unremarkable inferior olives at the age of 7 years, but development of hypertrophic olivary degeneration needs to be carefully followed-up in correlation with first clinical signs of ataxia and disturbance of speech.

Consistent with previous reports [1-6], cerebellar atrophy was found in our patients, but no increased signals in the dentate nucleus (Figure 2).

The *TTC19* mutations identified in our patients differ considerably from the mutations previously reported. So far, only nonsense and frameshift mutations due to small deletions and duplications have been described [1-5]. Both kinds of mutations are regarded deleterious as they result in a premature stop codon subjecting the generated mRNA to nonsense-mediated mRNA decay [33] or the translation of substantially truncated proteins.

For the first time, we identified two novel missense mutations (c.971 T > C; p.Leu324Pro and c.554 T > C; p.Leu185Pro) in three of our patients (patients 1, 3, and 4), thus expanding the mutational spectrum of patients with *TTC19* mutations. The quantification of the TTC19 cDNA in these patients showed a similar amount compared to controls, which indicates a stable transcript for these missense mutations. Both mutations are localized within tetratricopeptide repeats that are conserved within TTC19 proteins. The replacement of leucine for proline, which was found in both missense mutations, is expected to affect the helical structure of these TPR domains. Notably, the missense mutation in patient 1 was associated with an early and previously not reported onset of disease in terms of neonatal lactic acidosis and lactic acidosis during intercurrent febrile infections.

The nonsense mutation c.656 T > G (p.Leu219*), which was found in patient 2 has been previously described in two families [1], and is the only recurrent mutation in *TTC19* so far.

So far, the biochemical indication for TTC19 deficiency has been MRC complex III deficiency [1-3,5,6], either isolated [2,3] or in combination with other MRC complexes [5]. This finding is in agreement with our patients 1 and 2. In patient 3, however, activity of MRC complex III was normal in two independent muscle biopsies analyzed in different metabolic centers. In this patient, diagnosis of TTC19 deficiency would have been missed if mutational or western blot analysis was only taken in account in patients with MRC complex III deficiency. To our knowledge, this is the first patient with TTC19 deficiency showing a normal MRC complex III activity. Furthermore, the amount of complex III and its subunits (core 1, core 2, and Rieske protein) in Blue Native and SDS electrophoresis was found to be normal in patients 1, 2, and 3 (fibroblasts, muscle), which complicates the diagnosis of TTC19 deficiency.

## Conclusions

TTC19 deficiency is a rare but maybe underdiagnosed mitochondrial disorder. We present four pediatric patients from 3 unrelated families 3 of them being severely affected. For the first time, we describe homozygous missense mutations in TTC19 patients, as well as one patient presenting with episodes of severe lactic acidosis, and one patient with normal MRC complex III activity. Thus, we expand the genetic, clinical, and biochemical spectrum of the disease. Reviewing the literature, we delineate very characteristic clinical, biochemical, and neuroimaging features, which should alert clinicians to the possibility of the diagnosis TTC19 deficiency.

## Abbreviations

CSF: Cerebrospinal fluid
COX: Cytochrome c oxidase
MR: Magnetic resonance
MRC: Mitochondrial respiratory chain
PDH: Pyruvate dehydrogenase
PEG: Percutaneous endoscopic gastrostomy
SDH: Succinate dehydrogenase
TPR: Tetratricopeptide repeat
